# Microtubules: A Key to Understand and Correct Neuronal Defects in CDKL5 Deficiency Disorder?

**DOI:** 10.3390/ijms20174075

**Published:** 2019-08-21

**Authors:** Isabella Barbiero, Roberta De Rosa, Charlotte Kilstrup-Nielsen

**Affiliations:** Department of Biotechnology and Life Sciences, (DBSV), University of Insubria, Via Manara 7, 21052 Busto Arsizio (VA), Italy

**Keywords:** CDKL5, microtubules, neuronal morphology, +TIP, CLIP170, EB1–3, MAP1S, microtubule-targeting agents, pregnenolone

## Abstract

CDKL5 deficiency disorder (CDD) is a severe neurodevelopmental encephalopathy caused by mutations in the X-linked *CDKL5* gene that encodes a serine/threonine kinase. CDD is characterised by the early onset of seizures and impaired cognitive and motor skills. Loss of CDKL5 in vitro and in vivo affects neuronal morphology at early and late stages of maturation, suggesting a link between CDKL5 and the neuronal cytoskeleton. Recently, various microtubule (MT)-binding proteins have been identified as interactors of CDKL5, indicating that its roles converge on regulating MT functioning. MTs are dynamic structures that are important for neuronal morphology, migration and polarity. The delicate control of MT dynamics is fundamental for proper neuronal functions, as evidenced by the fact that aberrant MT dynamics are involved in various neurological disorders. In this review, we highlight the link between CDKL5 and MTs, discussing how CDKL5 deficiency may lead to deranged neuronal functions through aberrant MT dynamics. Finally, we discuss whether the regulation of MT dynamics through microtubule-targeting agents may represent a novel strategy for future pharmacological approaches in the CDD field.

## 1. Introduction

The cyclin-dependent kinase-like 5 (*CDKL5*) gene is known since 1998, when it was identified in a transcriptional mapping approach to identify disease-causing genes in the Xp22 region [1]. However, the gene caught significant attention only in 2003, when translocations interrupting *CDKL5* were identified in two female patients with severe X-linked infantile spasms and intellectual disability [2]. Rapidly afterwards, in 2004, more reports described missense and frame-shift mutations in various positions of *CDKL5* in individuals with clinical manifestations, ranging from atypical Rett syndrome (RTT) to autism [3,4,5]. In the following reports, mutations in *CDKL5* were commonly identified in patients clinically diagnosed with the early seizure variant of RTT and it was only in 2013 that the pathology associated with *CDKL5* deficiency was established as an independent clinical entity referred to as CDKL5 disorder [6] or, lately, as CDKL5 deficiency disorder (CDD). The first clinical manifestation of CDD is normally treatment-resistant seizures that appear within the first three months after birth [7]. Moreover, CDD patients are characterised by developmental delay, intellectual disability, gross motor impairment, strong hypotonia, sleep disturbances, gastrointestinal problems, breathing problems and hand stereotypies. Some of these symptoms overlap with those of RTT, and the main distinction between the two pathologies is the absence of a period of regression, which is among the diagnostic criteria for RTT [8], in patients with *CDKL5* mutations.

The clinical manifestations linked to *CDKL5* mutations underscore the importance of the encoded serine/threonine kinase for brain functions. Studies aimed at understanding the molecular pathways controlled by CDKL5 and the neuronal and neuroanatomic consequences associated with its deficiency have in the last years provided crucial information of CDKL5 functions. Importantly, very recently, reports from more laboratories converge on a functional link between CDKL5 and microtubules (MTs) [9,10,11,12]. MTs, together with actin and intermediate filaments, constitute a highly dynamic architectural element of neurons and are fundamental for intracellular cargo transport and for the accommodation of structural neuronal changes that underlie synaptic plasticity [13]. Various brain disorders have been linked to mutations in genes that control MT-related processes, and the pharmacological targeting of MTs or the associated proteins are currently being tested in preclinical and clinical studies [14,15].

We will here review the recent findings regarding CDKL5 functions with particular focus on its MT-related functions and the possible translational value of these findings for CDKL5-related disorders.

## 2. Microtubules

**Figure 1:**
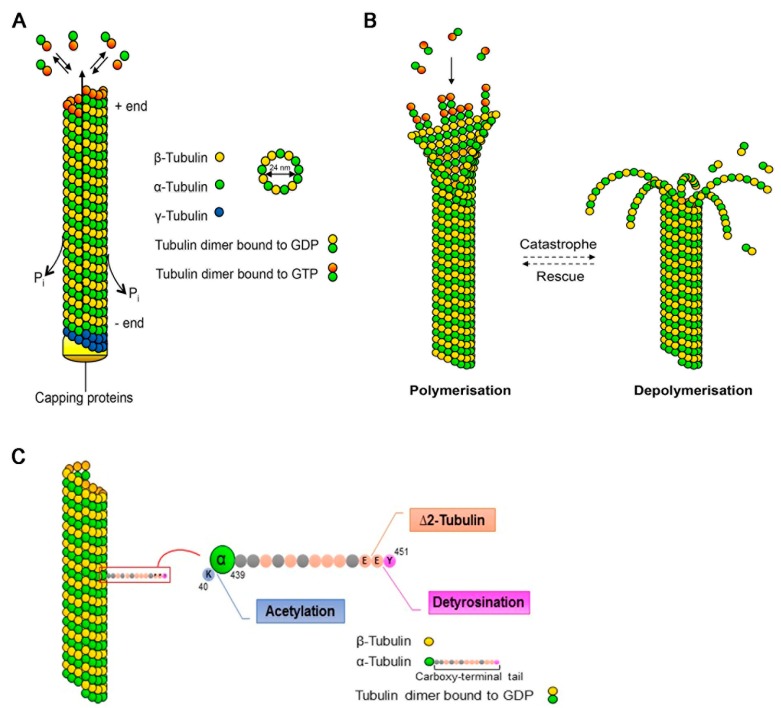
Microtubule dynamics and post-translational modifications. (**A**) Microtubules (MTs) are composed of α- and β-tubulin heterodimers that assemble to linear proto-filaments that associate laterally, forming the hollow MT cylinder. Assembly, which is nucleated from the γ-tubulin ring, occurs in a polarised fashion with the β-tubulin subunit oriented towards the growing plus-end. Newly-incorporated tubulin is bound to guanosine triphosphate (GTP) that gets rapidly hydrolysed upon polymerisation, generating guanosine diphosphate (GDP)-bound tubulin along the MT lattice. (**B**) MTs are dynamic structures undergoing cycles of growth (polymerisation) and shrinkage (depolymerisation). The switch from growth to shrinkage is termed catastrophe, whereas rescue indicates the switch from shrinkage to growth. (**C**) The α-tubulin subunit can be acetylated on lysine (K) 40, which is located on the inner side of the MT. The C-terminal tail of α-tubulin projects away from the MT lattice and undergoes various post-translational modifications. The extreme tyrosine residue (Y) can be enzymatically removed, generating detyrosinated α-tubulin, which normally correlates with long-lived MTs. Further removal of the penultimate glutamate (E) generates ∆2-tubulin.

Microtubules are constituted by dimers of α- and β-tubulin, polymerising in a head-to-tail fashion into proto-filaments that assemble laterally to form polarised tubular structures with so-called plus- and minus-ends exposing β- and α-tubulin, respectively [13] (Figure 1A). Assembly requires β-tubulin to be in its guanosine triphosphate (GTP)-bound form; the rapid hydrolysis into guanosine diphosphate (GDP) upon incorporation into the MT lattice generates a GTP-cap at the plus-end, favouring further polymerisation. In most cells, MTs are nucleated from the MT-organising centre (MTOC) with the minus-end of the MTs being embedded and stabilised in the γ-tubulin ring and the plus-ends radiating into the cytoplasm. Neuronal MTs, however, are rapidly released by MT-severing proteins and then transported into the neurites as short polymers. Further, in neurons, MT nucleation can also occur independently of the MTOC [16], with the minus-ends being capped and stabilised by specific proteins. The highly-polarised nature of neurons is mirrored in the organisation of the MT array: in the axon, MTs are uniformly oriented with the plus-ends facing the tip of the axon, while in dendrites they are of mixed polarity with both minus- and plus-ends pointing distally.

MTs are highly dynamic structures that undergo cycles of rapid growth and disassembly in a process defined as dynamic instability [13] (Figure 1B). The switch from growth to disassembly (or shrinkage) is called “catastrophe”, while the reverse is termed “rescue”. This dynamic instability, which occurs mainly at the MT plus-ends, provides a rapid reorganisation of the cytoskeleton that is necessary for many cellular functions, such as cell division and migration and formation of cell polarity. Various factors participate in the control of dynamic instability such as the fine regulation of MT-associated proteins, among which the plus-end tracking proteins (+TIPs) play a fundamental role [17], and the post-translational modifications (PTMs) of α- and β-tubulin that constitute the so-called “tubulin code” [18].

Tubulin PTMs present different allocations on the α/β-tubulin heterodimer with respect to their position on the MT lattice: detyrosination and deglutamylation take place on the C-terminal tail of tubulin that projects away from the MT lattice and provides interaction sites for the MT-associated proteins. Conversely, acetylation of α-tubulin on lysine 40 is found on the inside of the MT lumen (Figure 1C) [18]. Newly-synthesised α-tubulin generally contains a C-terminal tyrosine residue that undergoes enzymatic cyclic removal and re-addition. Detyrosinated MTs are normally considered stable and long-lived, even if the modification per se does not influence MT stability but rather alters the anchorage point for specific proteins. The irreversible removal of the penultimate glutamate from the primary sequence of α-tubulin, generating ∆2-tubulin, prevents α-tubulin re-tyrosination and represents a hallmark of stable MTs. Acetylation of α-tubulin on lysine 40 that, as mentioned, is located within the lumen of the MT lattice is synonymous of stable MTs. Although the causal link between tubulin PTMs and MT stability remains largely enigmatic, the consequences of their disruption can be fatal, as illustrated by animal models carrying inactivating mutations in genes encoding the modifying enzymes [19].

As mentioned, dynamic instability is largely influenced by a plethora of proteins that bind MTs. Classical MT-associated proteins (MAPs) constitute an important group of proteins that bind along the MT lattice, influencing its polymerisation, stability and bundling. MAP1 family proteins, such as MAP1A, MAP1B and MAP1S, are high molecular weight complexes formed of heavy and light chains (LC) [20]. The capacity of these proteins to bind both MTs and actin suggests that they might simultaneously bind and cross-link these two cytoskeletal components. The MAP2 family includes MAP2 and Tau that accumulate in neuronal dendrites and axons, respectively, where they promote MT stability [21]. 

The +TIP family comprises another large group of diverse proteins that share the common feature of associating with the growing MT plus-end, where they control MT dynamics and functioning [22]. The plus-end tracking behaviour of these proteins can be visualised in live-imaging studies where the binding of the fluorescently tagged proteins to the growing MTs appears as comet-like dashes [23]. The end-binding proteins (EBs) form the central hub of the +TIPs; indeed, they provide the attachment site for other +TIPs thanks to their autonomous binding to MT plus-ends [24]. The EB family comprises EB1–3 that bind MTs through their calponin homology (CH) domain in the Ν-terminus, while they engage in specific protein interactions through C-terminal regions. The cytoskeleton-associated protein Gly-rich (CAP-Gly) domain proteins, including the cytoplasmic linker proteins (CLIP) CLIP170, CLIP115, and p150-Glued, constitute another important group of +TIPs that bind MT plus-ends indirectly via EBs but also by directly recognising the C-terminal EEY/F (glutamate-glutamate-tyrosine/phenylalanine) motif of α-tubulin [19,25,26]. Consistently, detyrosinated α-tubulin is deprived of the binding site for the CAP-Gly proteins. Another large group of +TIPs, including proteins like the CLIP-associated proteins (CLASP), the tumour suppressor adenomatousis polyposis coli protein (APC), p140Cap, and some kinesins (KIF), is characterised by an SxIP (serine-any amino acid-isoleucine-proline) motif through which they bind the EBs [17]. 

Functionally, the +TIPs can affect MT dynamics in opposing ways with the EBs suppressing the frequency of MT catastrophes and the CLIPs promoting MT rescue [22]. The +TIPs play an essential role in MT-related functions by mediating reciprocal interactions between the MT plus-ends and the cell cortex, where several of these proteins interact directly or indirectly with the actin cytoskeleton. This allows a subset of MTs to be selectively stabilised at specific sites under the cell membrane creating tracks for the delivery of proteins to the cell periphery and pulling forces on the MT network. In this way, the +TIPs are involved in various cellular processes including mitosis, cell migration and morphology [27]. Regarding neurons, various stages of neuronal development such as neuronal migration, axonal growth and dendritic spine maturation are strictly controlled by the +TIPs.

The importance of the proper regulation of the MT network for neuronal functions has been demonstrated through the pharmacological manipulation of MT dynamics. Indeed, axon formation can be artificially induced treating unpolarised neurons with the MT-stabilising drug, taxol [28]. On the other hand, both dendritic outgrowth and the maintenance of mushroom-headed spines were reduced through the inhibition of MT assembly [29,30]. Further, mutations in various genes encoding proteins that regulate MT dynamics can cause defects in neuronal migration and connectivity and have been linked to neurodevelopmental disorders characterised by intellectual disability and autistic features [31]. 

As mentioned, several links have recently been established between CDKL5 and MTs. Indeed, CDKL5 has recently been found to interact with the two +TIPs, CLIP170 and EB2 [9,10,11]. The latter was identified through a chemical genetic approach as a CDKL5 substrate and was thoroughly characterised as a bona fide phosphorylation target of CDKL5 in vitro and in vivo; the functional consequences of this event are still unknown, however [11], and neuronal functions of EB2 have not been described so far. CLIP170 was related functionally to CDKL5 through the identification of IQ motif containing GTPase activating protein 1 (IQGAP1) as a novel CDKL5 interactor [9]. IQGAP1 is an actin-binding protein that binds CLIP170 in a Rac1-dependent manner, facilitating the transient capture of MTs at specific cortical sites under the cell membrane [32,33]. Further, MAP1S, the less characterised member of the MAP1 family proteins, was identified as a substrate of CDKL5 through two different approaches providing strong support of a functional role of this association [11,12].

Since the discovery of the first patients with *CDKL5* mutations, CDKL5 functions have been studied in vitro and in vivo in proliferating cells, primary neurons, in mouse models carrying inactivating deletions of the *Cdkl5*-gene and in human induced pluripotent stem cells (iPSC)-derived neurons [34]. The overall picture emerging from these studies indicates that CDKL5 deficiency impacts cell proliferation, neuronal migration, and various aspects of neuronal morphology that are all processes that are strictly influenced by MT dynamics. These findings, demonstrating that CDKL5 functions converge on MT dynamics, may, therefore, provide a key to understand the mechanisms underlying CDD in more details.

## 3. Neuronal CDKL5 Related Defects

### 3.1. Axon Formation

**Figure 2:**
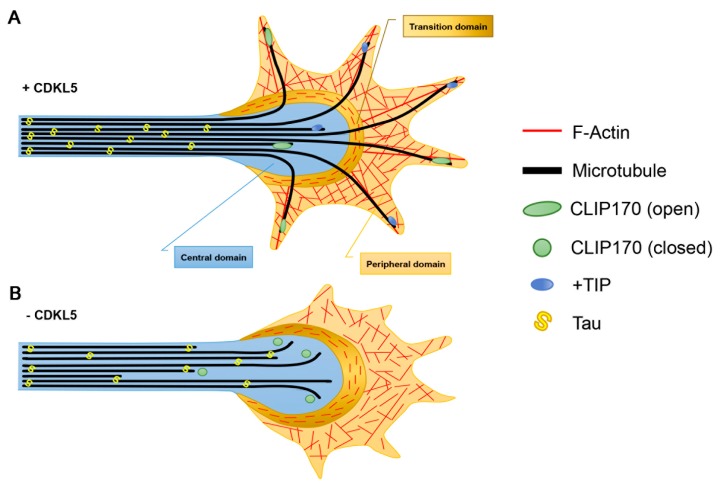
The effect of CDKL5 deficiency on microtubule dynamics in axonal growth cones. (**A**) In the axon, microtubules (MTs) are organised in a polar fashion with the plus-ends oriented distally. The central domain, which is contiguous with the axonal shaft, is rich in MTs that are bound by plus-end tracking proteins (+TIPS) including CLIP170. The peripheral domain is rich in actin that extends into the filopodia. The transition zone, lying in between these two domains, contains antiparallel actin arcs that form a barrier against the invasion of MTs into the peripheral domain. (**B**) In the absence of CDKL5, CLIP170 is in its closed conformation and is less associated with the MT plus-ends impacting their capacity to engorge into the actin rich area and leading to cessation of axonal outgrowth.

In primary low-density cultures of hippocampal neurons, CDKL5 controls axon specification and outgrowth [35,36]. Axon elongation relies on the formation of stable MTs in the axonal shaft. These MTs form bundles covered with the axon specific MAP, Tau, and are organised in a polarised fashion, with the plus-ends oriented towards the peripheral tip. Such MT organisation generates tracks that facilitate the transport of proteins and organelles, thus providing the building blocks for new axonal segments. The axonal tip is characterised by a fan-shaped, motile structure, the growth cone, that probes the extracellular environment. In this way axon elongation and steering is directed in a process that depends on the complex remodelling and reorganisation of MTs and actin (Figure 2). The growth cone comprises the central region, enriched in MTs, and the peripheral domain, which is mainly composed of filamentous actin (F-actin); the two regions are separated by the transition zone in which actin arcs form a barrier against the extension of MTs into the peripheral zone [37]. Few MTs are capable of escaping the central domain and reach the growth cone periphery where they, thanks to the +TIPs, can couple to F-actin and associate with specific sites at the growth cone cortex. In particular, the +TIP CLIP170 accumulates in the axonal growth cone by binding to tyrosinated tubulin [19,38]. Interfering with CLIP170 expression impairs axon formation in primary neurons. This is accompanied by a reduced capacity of MTs to penetrate the actin arcs and protrude into the peripheral domain [39]. CDKL5 deficiency reduces the association of CLIP170 to MTs in proliferating cells and in the axonal growth cone of *Cdkl5*-KO neurons, which in both cases is accompanied by altered MT dynamics [9,40]. The binding of CLIP170 to MTs occurs when the protein is in its open active conformation whereas the intramolecular interaction between its terminal CAP-Gly domain and zinc-knuckle motifs causes a closed inactive conformation [41]. Interestingly, the neurosteroid pregnenolone (PREG), which induces the open conformation of CLIP170 through its direct binding to the protein [42], is capable of normalising the MT-binding of CLIP170 and restore axon specification and elongation in CDKL5 deficient neurons [9]. It thus seems likely that CDKL5 is involved in maintaining CLIP170 in its active conformation. The switching between the open and closed conformation of CLIP170 is known to depend on specific phosphorylation events. Whether CDKL5 regulates CLIP170 in a phosphorylation-dependent manner is still not known.

In the past, CDKL5 was proposed to affect axon formation through its interaction with Shootin1 [35]. Shootin1 is a key determinant for axon specification [43]; it was found to act as a clutch molecule that produces the traction force for axon outgrowth by coupling the retrograde flow of F-actin and cell adhesions [44]. Considering the novel link with CLIP170 it, thus, appears that CDKL5 may influence axon formation in different ways.

### 3.2. Dendritic Arborisation 

CDKL5 deficiency was first shown, through silencing studies, to influence dendritic arborisation in vitro in primary cultures of rat cortical neurons and in vivo in migrating neurons derived from silenced neural progenitor cells [45]. Such defect has later been confirmed in cortical and hippocampal pyramidal neurons in *Cdkl5*-KO brains [46,47,48] and reproduced in vitro in *Cdkl5*-KO primary hippocampal neurons [36,40]. The penetrance of the phenotype in vitro may depend on the experimental conditions since contrasting data have been reported [11].

The control of CDKL5 on dendrite morphology was found to occur through Rac1, which is a critical regulator of actin remodelling. Recombinant CDKL5 was capable of pulling out Rac1 from neuronal lysates, an interaction that was further stimulated upon prior neuronal stimulation with brain-derived neurotrophic factor (Bdnf). Further, Bdnf activation of Rac1 was impaired in CDKL5 silenced cells, thus placing Rac1 downstream the kinase [45]. The exact mechanism through which CDKL5 activates Rac1 is unknown; however, it may be relevant to consider that CDKL5 controls the stability of the ternary IQGAP1/CLIP170/Rac1 complex [9]. IQGAP1 is widely implicated in Rac1/cdc42 signalling [49]. Indeed, IQGAP1 maintains the two small GTPases in their active GTP-bound state and acts as a scaffolding platform for Rac1/cdc42 signalling by recruiting their downstream effector proteins. Further, CLIP170 and IQGAP1 were reported to cooperate in controlling dendrite morphology, apparently by bridging MTs and the actin cytoskeleton [50]. Reduced CLIP170 or IQGAP1 levels led to the formation of a less complex dendritic arbor, a phenotype that resembles the one linked to CDKL5 deficiency and that could be prevented through the pharmacological stabilisation of F-actin. CDKL5 may, thus, control the cytoskeleton dynamics involved in dendrite morphology by allowing the formation of the protein complex mediating Rac1 activation and bridging the MT and actin networks.

**Figure 3:**
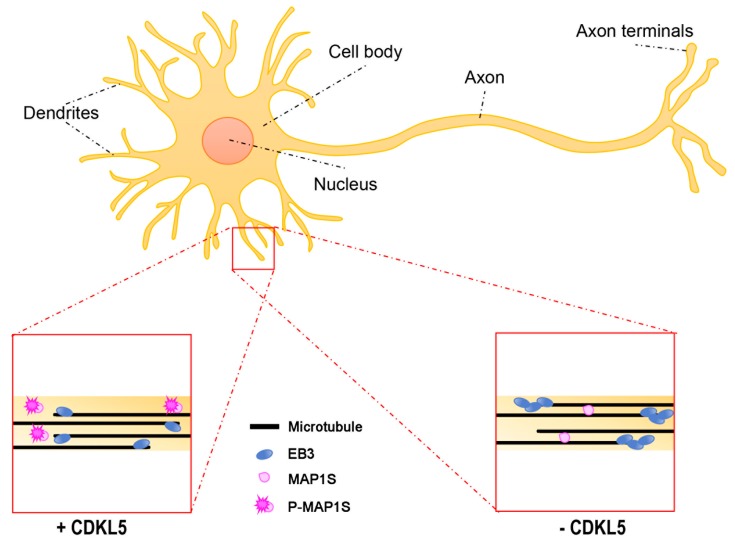
The effect of CDKL5 deficiency on dendritic microtubule dynamics. In dendrites, microtubules (MTs) have mixed orientations with the plus-ends oriented both proximally and distally. CDKL5 phosphorylates MAP1S inhibiting its binding to MTs (lower left). In the absence of CDKL5 (lower right), the binding of MAP1S to MTs is accompanied by increased life-time of EB3 binding to the plus-ends.

Another possible way in which CDKL5 regulates MT dynamics during dendritic arborisation may be represented by the phosphorylation on MAP1S, recently identified as a bona fide substrate of CDKL5 [11,12] (Figure 3). When phosphorylated on two serines (Ser786 and Ser812), located within the LC, MAP1S LCs have reduced affinity for MTs and appear to be more soluble. Interestingly, overexpression of phospho-defective MAP1S LCs in neurons forced the dendritic tips to settle in an aberrant looped conformation. In line with the capacity of MAP1 family members to increase MT stability [51], functional studies suggested that the increased binding of MAP1S to MTs in the absence of CDKL5 leads to less dynamic MT plus-ends. Indeed, EB3-GFP (green fluorescent protein) comets, the fluorescence of which over time is a measure of MT dynamicity, are longer lived in *Cdkl5*-KO neurons, but are restored to normal life-times upon MAP1S silencing [11].

### 3.3. Dendritic Spines

Various studies in vitro and in vivo converge on a role of CDKL5 in controlling dendritic spine morphology and excitatory functions. Neurons devoid of CDKL5 are characterised by an increased number of filopodia-like protrusions and a reduced number of mushroom-shaped spines [52,53]. The enlarged spine heads, which distinguish mushroom-shaped spines, correlate with increased synaptic strength. Various neurologic conditions characterised by cognitive dysfunctions and autistic features have been linked to altered density and shape of dendritic spines [54]. CDKL5 is readily detectable in dendritic spines, where its synaptic accumulation is regulated both by the dendritic targeting of its mRNA, which undergoes local activity-dependent translation, and through its interaction with post-synaptic density protein 95 (PSD95) [52,55,56]. CDKL5 is, thus, likely to influence directly various proteins involved in excitatory neurotransmission. Accordingly, CDKL5 affects the expression or phosphorylation of the post-synaptic markers PSD95, Homer, the α-amino-3-hydroxy-5-methylisozasole-4-propionic acid (AMPA)-receptor subunit GluA2, and NGL1 [52,56,57,58]. So far, we have very little information whether the neuroanatomical and molecular alterations in CDD mouse models are present also in the patients. However, reduced membrane levels of GluA2, which have been observed in vitro in neurons silenced for CDKL5 expression and in vivo in the hippocampus and perirhinal cortex of two mouse models of CDD, were recently confirmed in post-mortem brains from two CDD patients [58,59,60], suggesting the relevance of at least some CDKL5-related defects for the human pathology.

**Figure 4:**
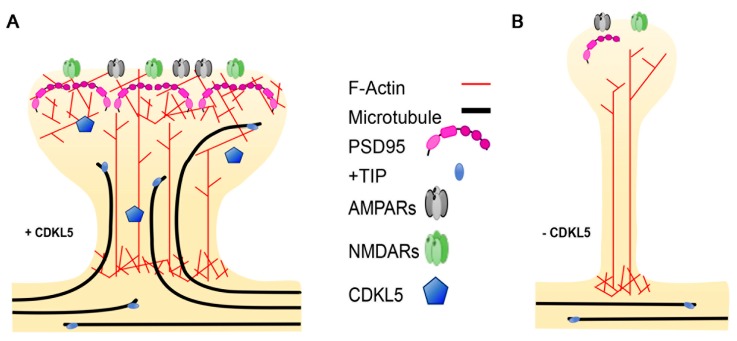
The effect of CDKL5 deficiency on dendritic spine morphology. (**A**) Dendritic spines, which are enriched in actin, are invaded by microtubules (MTs) upon neuronal activation. The plus-end tracking proteins (+TIP), bound to the MT plus-ends, interact with actin-binding proteins facilitating the invasion. (**B**) In the absence of CDKL5, MTs are less dynamic and fail to invade the spines, impacting the activity-dependent structural changes that lead to the formation of mushroom-shaped spines.

Plasticity-related changes in spine morphology are widely known to rely on remodelling of the actin cytoskeleton, which is highly enriched in dendritic protrusions [61]. MTs were for many years ignored as an integral part of dendritic spines; however, live imaging studies of fluorescently-tagged EB3, marking dynamic MTs, combined with other techniques, have allowed demonstrating that MTs do actually polymerise into dendritic spines in a rapid and transient manner [30,62,63] (Figure 4). The incursion of MTs into the spines is activity-dependent and contributes to the formation of mature dendritic spines. Actually, nocodazole-mediated inhibition of dynamic MTs impedes the Bdnf-induced structural changes that leads to the maturation of filopodia-like protrusions into mushroom-shaped spines [63]. The +TIP EB3 plays an active role in MT-dependent structural plasticity; actually, its silencing leads to a reduced number of mushroom-shaped spines. Paralleling the importance of a cross-talk between MTs and the actin cytoskeleton in the axonal growth cone, EB3 was found to provide a link to the actin cytoskeleton in the spines. Indeed, interactions between MT-bound EB3 with p140Cap and the actin stabilising factor cortactin is likely to create local signalling events that allow MT-actin communication in “activated” spines [30]. Interestingly, the actin–MT cooperation seems, in turn, to be enhanced by neuronal network activation; indeed, very recent data suggest that MT invasion occurs in specific spines through N-methyl-D-aspartate (NMDA)-receptor-mediated calcium influx that leads to actin remodelling at the base of the spine, thus allowing the targeting of the MTs [64].

Regarding a possible role of CDKL5 in regulating spine morphogenesis through MT-related events, it is tempting to speculate that more +TIPs may be involved in regulating spine morphogenesis and functions creating other distinct links between MTs and the actin cytoskeleton. This would allow an expansion in the repertoire of possible signalling events underlying structural plasticity. While EB2 is broadly expressed in the neuronal soma, its levels are very low in the post-synaptic compartment [65]. Accordingly, CDKL5-dependent phosphorylation of EB2 on serine 223 can readily be detected in the proximal dendrites of patient iPSC-derived neurons [11]. The presence of CLIP170 in dendritic spines has so far not been reported. However, IQGAP1, which interacts with both CDKL5 and CLIP170, is present in spines [66], where it regulates spine morphogenesis [67], and *Iqgap1*-KO mice display cognitive defects and impaired long-term potentiation (LTP) [68]. IQGAP1 interacts with various post-synaptic proteins such as the AMPA-receptor GluA4, PSD95 and the NMDA-receptor complex [68,69]; it is, thus, intriguing to speculate that its interaction with CLIP170 might serve a role in regulating spine formation and functioning. Of further interest, disks large homolog 5 (DLG5), which was identified as a cellular substrate of CDKL5 [12], is an important regulator of dendritic spine formation [70]. DLG5 is member of the membrane-associated guanylate kinase (MAGUK) proteins and appears to control spine formation by facilitating the localisation of N-cadherin to the cell membrane. A possible link between DLG5 and MTs can be hypothesised based on its interaction with MARK3 (MT affinity regulating kinase 3), a kinase that controls cell polarity through the phosphorylation of specific MAPs, thus regulating their affinity for MTs [71].

Considering that MTs constitute fundamental tracks for the motor-based transport of cargoes, their invasion into dendritic spines is likely to provide a way of transporting cargoes into and out of these structures. The nature of which cargoes enter spines through MTs is still not clear. However, synaptotagmin has been found to be transported in a kinesin-dependent manner into dendritic spines [72]. Further studies will be relevant to analyse the contribution of altered MT dynamics to CDKL5-related spine defects and which cargoes might be influenced by CDKL5 deficiency.

## 4. Non-Neuronal CDKL5-Related Defects

The role of CDKL5 in regulating MT dynamics is consistent with our previous report that described CDKL5 as a centrosomal protein [10]. The centrosome is a central component of the cytoskeletal structure and consists of a scaffold core containing a large number of proteins, including γ-tubulin and the γ-tubulin ring complex (γ-TuRC) that typically surrounds a pair of cylindrical centrioles enclosed in the amorphous pericentriolar material (PCM) [73]. The centrosome is the primary MT-organising centre of the cells and, therefore, participates in fundamental cellular functions, such as cell motility, migration, polarity [74,75] and in the assembly and organisation of the mitotic spindle [76,77]. CDKL5 silencing causes an increase in the number of defective multipolar spindles, which is associated with polyploidisation and centrosome accumulation [10]. It is interesting to note that CDKL5 was recently found to interact with and phosphorylate the centrosomal protein CEP131 on serine 35 [12]. The functional role of this phosphorylation event was not further explored; however, silencing of CEP131 has been reported to cause centriole amplification and chromosomal instability [78], a phenotype that overlaps the one caused by CDKL5 deficiency [10]. Furthermore, CDKL5 and CEP131 share a common role in ciliogenesis [79,80]. The primary cilium is a eukaryotic organelle that is present on most quiescent and differentiated cells; it originates from a centrosome-derived structure, the basal body, and is composed by a central MT structure, the axoneme. The primary cilium acts as a cellular antenna that transmits signals from the extracellular environment to the inner site of the cell, thereby contributing to the regulation of numerous cellular processes such as proliferation, differentiation, migration and cell polarity [81]. Interestingly, defects in cilia morphogenesis and functioning have been associated with a large list of heterogeneous genetic disorders, known as ciliopathies [82]. Among the various clinical manifestations linked to ciliary dysfunctions, some share an overlap with those of CDD such as developmental delay, epilepsy, cognitive dysfunction and aberrant respiration. Cilium length appears to be influenced by CDKL5 levels [79,83]; a further understanding of how CDKL5 dysfunction impacts ciliary functions and the contribution of CEP131 and MT dynamics may pave the way for new perspectives in the CDKL5 field.

## 5. Possible Functional Outcome of Specific Pathogenic CDKL5 Mutations

The above studies linking CDKL5 to MT-related functions have been addressed in cells or tissues silenced for CDKL5 or with no expression of the protein. This reflects well the current view that CDD is caused by the absence of functional CDKL5 or by the expression of hypomorphic derivatives. Indeed, CDD-causing mutations in *CDKL5* include missense mutations, nonsense, frame-shift and splicing variants, and large genomic deletions [84]. The pathogenic missense mutations, which have been identified so far, fall almost exclusively within the catalytic domain of the protein. Functional studies based on the exogenous expression of mutated CDKL5 derivatives suggest that amino acid substitutions within the kinase domain impact negatively its catalytic activity [79,85,86,87]. Although these studies have highlighted the impact of missense mutations on CDKL5 functioning, it needs to be underlined that only very few of these mutations have been studied; we therefore cannot exclude that other missense mutations may generate hypermorphic CDKL5 derivatives. Regarding the nonsense and frame-shift variants, nonsense-mediated decay of the mutated mRNA is likely to impede the expression of the encoded protein, thus acting as hypomorphic or null mutations [84,88,89]. 

The existing animal models of CDD have addressed the consequences of the absence of CDKL5 either in the whole body or in distinct brain areas [46,47,48,90]. Recently, studies were performed in a mouse line expressing the pathogenic R59X allele, but since this causes the absence of CDKL5, it can be considered a functional null [60,91]. Although the generation of CDD mouse models has been extremely useful to deeply understand the roles of CDKL5, an important aspect that still remains to be addressed is how the expression of pathogenic hypomorphic CDKL5 derivatives affects neuronal functions. Hypothetically, such derivatives could behave as dominant negatives and interfere with the normal function of CDKL5 interactors, such as the MT-interacting proteins. Of relevance, CDKL5 localisation and cilium length is influenced by the expression of CDKL5 derivatives containing CDD-causing missense mutations that have not yet been characterised for the catalytic activity [79]. While the field is awaiting such animal models, primary fibroblasts and iPSC-derived neurons obtained from CDD patients will be very informative. Finally, it must be remembered that large genomic duplications, including *CDKL5*, have been identified in individuals with varying degrees of macrocephaly and learning disabilities [92], suggesting the relevance of analysing the effect of increased CDKL5 levels on MT-related functions.

## 6. Therapeutic Relevance of CDKL5-Dependent MT Defects

Alterations of the MT cytoskeleton have been linked to neurodegenerative and neurodevelopmental disorders such as schizophrenia and autism and major depression disorder [15,93]. Efforts have, therefore, been put into the development of therapeutic strategies targeting the neuronal cytoskeleton for these disorders [14,94]. The pharmacological stabilisation of MTs with the consequent block of mitosis has for decades been used for cancer treatment; the possibility of repositioning these drugs has been considered for brain disorders. MT-stabilising drugs were initially considered for neurodegenerative disorders that are characterised by compromised tau functions and altered MT structure and axonal transport [94]. Taxol (paclitaxel) promotes MT stability by binding within the MT lumen and increasing the lateral interactions between adjacent proto-filaments [95]. When considering the use of MT-stabilising drugs for brain disorders, it is fundamental to consider their capacity to pass the blood–brain barrier and their possible neuropathy side effects. While proof-of-principle studies supported the beneficial effect of taxol-mediated MT stabilisation on a mouse model presenting tau pathology in spinal motor neurons [96], this drug has a limited bioavailability in brain since it does not cross the blood–brain barrier. As taxol, Epothilone D (EpoD) binds and stabilises MTs, but it has the advantage of being brain penetrant, thus holding a stronger therapeutic potential for brain disorders [97]. EpoD has been tested in animal models of tauopathies where doses much lower (1/30) than those used in cancer trials were found to be effective in stabilising MTs and to be capable of reducing cognitive deficits present in these models [98,99]. While EpoD and other drugs with similar MT-stabilising activities may possess interesting therapeutic potentials for patients with neurodegenerative diseases, their clinical application for young patient groups, as for example CDD, is more difficult to imagine. Drugs that bind MTs directly will impact MT stability in a non-specific manner and may, thus, have broad and non-advantageous effects on neurodevelopmental processes that are still ongoing in young patients. The use of drugs that target MT-binding proteins may act more specifically and, therefore, represent valid therapeutic options. An interesting molecule is represented by NAP/davunetide, an eight amino acid peptide that is derived from the activity-dependent neuroprotective protein (ADNP) [100]. ADNP is a versatile protein interacting with SWI/SNF chromatin remodelling complexes and with the +TIPs EB1 and EB3 [101,102]. Mutations in *ADNP* have been linked to autism and intellectual disability, and *Adnp* haploinsufficiency in mice is linked to altered axonal transport and dendritic spine alterations [103]. The NAP peptide interacts with the EB-proteins and promotes axonal transport and MT stability [104]. In pre-clinical studies in mouse models of various neurodegenerative diseases NAP shows neuroprotection and improves cognitive defects [105]. In clinical studies, intra-nasal administration of NAP led to improved cognitive skills in Alzheimer’s disease patients [106] and daily activities in schizophrenic patients [107]. 

The possibility of targeting of proteins that have been functionally linked to CDKL5 might hold an even higher therapeutic potential for CDD. Interestingly, the neuroactive steroid PREG was found to directly bind CLIP170, inducing its open active conformation, as well as MAP2 [108]. Notably, the treatment of CDKL5-silenced cells with PREG was capable of restoring the MT association of GFP-CLIP170 comets and neuronal defects linked to CDKL5 deficiency [9]. Treatment with PREG is known to enhance memory and cognition in rodents [109]. Further, clinical testing in humans indicated potential anti-depressive and anti-psychotic effects and a positive safety profile [110,111]. However, the clinical application of PREG is limited due to its short half-life and its conversion into other neuroactive steroids that altogether possess a broad range of activities acting at the genomic level and with numerous membrane-bound receptors [108]. The development and testing of synthetic derivatives with improved stability and that cannot be metabolised into the downstream steroids might represent a possibility for CDD. Of note, MAPREG is a synthetic PREG derivative that has anti-depressant activities in rat models of depression [112] and in the primate-like tree-shrew [113]. Like PREG, MAPREG/MAP4343 promotes MT dynamics apparently through its binding to MAP2. Through further studies it might be relevant to analyse the therapeutic potential of this or similar PREG derivatives on CDKL5-deficient neurons.

To conclude, the last findings of CDKL5 functions converging on MT dynamics are likely to be of great relevance not only for a deeper understanding of the neuronal dysfunctions linked to CDKL5 deficiency but also for the development of disease-modifying pharmacological approaches. Importantly, they may also lead to the identification of clinical diagnostic biomarkers. However, before considering MT-targeting drugs for clinical applications for CDD, we still need to understand in more detail how precisely CDKL5 deficiency affects MT dynamics.

## Abbreviations

+TIP	plus-end tracking protein
ADNP	activity-dependent neuroprotective protein
AMPA	α-amino-3-hydroxy-5-methylisozasole-4-propionic acid
APC	adenomatousis polyposis coli
Bdnf	brain-derived neurotrophic factor
CAP-Gly	cytoskeleton-associtated protein Gly-rich
CDD	CDKL5 deficiency disorder
CDKL5	cyclin-dependent kinase-like 5
CH	calponin homology
CLASP	CLIP-associated protein
CLIP	cytoplasmic linker protein
DLG5	disks large homolog 5
E	glutamate
EB	end-binding protein
EEY/F	glutamate-glutamate-tyrosine/phenylalanine
EpoD	Epothilone D
F-actin	filamentous actin
γ-TuRC	γ-tubulin ring complex
GFP	green fluorescent protein
GTP	guanine triphospate
GDP	guanine diphosphate
IQGAP1	IQ motif containing GTPase activating protein 1
iPSC	induced pluripotent stem cell
K	lysine
KIF	kinesin
LC	light chain
LTP	long-term potentiation
MAGUK	membrane-associated guanylate kinase
MAP	microtubule associated protein
MARK3	MT affinity regulating kinase 3
MT	microtubule
MTOC	microtubule organising centre
NMDA	N-methyl-D-aspartate
PCM	pericentriolar material
PREG	pregnenolone
PSD95	post-synaptic density protein 95
PTM	post-translational modification
RTT	Rett syndrome
SxIP	serine-any protein-isoleucine-proline
Y	tyrosine
